# Clinical characteristics and in-hospital outcome of heart failure in women: a single center registry from Egyptian cardiac care unit

**DOI:** 10.1186/s43044-019-0032-0

**Published:** 2019-12-09

**Authors:** Hala Mahfouz Badran, Marwa Ahmed Elgharably, Naglaa Faheem

**Affiliations:** Menoufia University, 55-ElGish street, Tanta, Gharbia P.O box 34 Egypt

**Keywords:** Heart failure, Gender difference, Women hospital mortality

## Abstract

**Background:**

This study represents figures from a cardiac care unit (CCU) of a university hospital; it describes an example of a tertiary academic center in Egypt and provides an epidemiological view of the female HF patients, their risk profile, and short-term outcome during hospitalization.

**Results:**

It is a local single-center cross-sectional observational registry of CCU patients 1 year from July 2015 to July 2016. Patient’s data were collected through a special software program. Women with evidence of HF were thoroughly studied.

Among the 1006 patients admitted to CCU in 1 year, 345 (34.2%) patients were females and 118 (34.2%) had evidence of HF, whereas 661 (65.7%) were males and 178 (26.9%) of them had HF. Women with HF showed 11.7% prevalence of the total population admitted to CCU. 72.7% were HFrEF and 27.3% were HFpEF. Compared to men, women with HF were older in age, more obese, less symptomatic than men, had higher incidence of associated co-morbidities, less likely to be re-admitted for HF, and less likely to have ACS and PCI. Valvular heart diseases and cardiomyopathies were the commonest etiologies of their HF. Women had more frequent normal ECG, higher EF%, and smaller LA size. There is no difference in medications and CCU procedures. While females had shorter stay, there is no significant difference in hospital mortality compared to male patients.

**Conclusions:**

Despite higher prevalence of HF in females admitted to CCU and different clinical characteristics and etiology of HF, female gender was associated with similar prognosis during hospital course compared to male gender.

## Background

Heart failure is a growing health challenge and among the major causes of death in developing countries along with the progression of the aging society, particularly in women [1, 2].

It has been generally accepted that female gender is associated with better survival (either crude and/or age-adjusted) compared with male gender in the broad spectrum of HF especially of a non-ischemic etiology, while other registries demonstrated no differences in the prognoses of male and female patients [3].

In the few studies from low-income countries, the gender distribution appears equal but age is much lower than in developed countries [4]. Etiologies have previously varied but recent studies suggest that HF in these countries increasingly shifts towards the pattern seen in developed countries with regard to risk factors, etiology, and comorbidity [5].

**Fig. 1:**
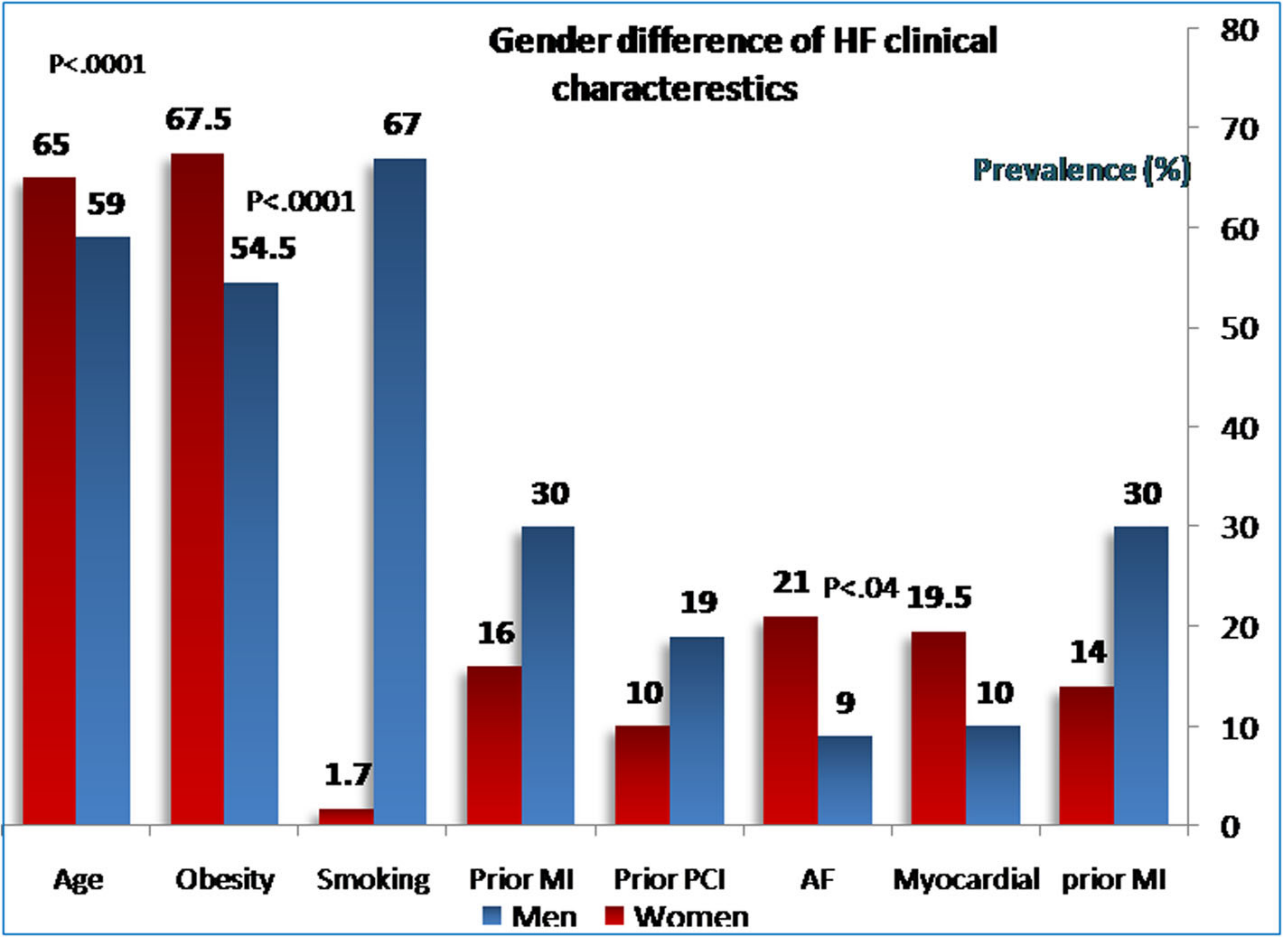
Gender difference of HF clinical characteristics

It continues to be mandatory to clarify, however, whether gender differences exist among Egyptian acute HF patients. Thus, in the present study, we addressed clinical characteristics and in-hospital management/outcomes of women using acute HF registry database (Fig. 1).

## Methods

### Baseline patient data

The present study is a single-center, prospective observational study. We enrolled 1006 consecutive patients > 18 years old with emergency admission to the CCU in university hospitals, a representative to tertiary academic center in Egypt between July 2015 and July 2016. Patients included in the study were those hospitalized due to worsening HF or new onset as the leading cause of admission. Patients in stages C–D HF were included in focused analysis [6]. The diagnosis of HF was based on the Framingham criteria for clinical HF [6]. The entry data were gathered by using an electronic data capture system; it included demographic data, etiology of HF, medical history, patient presentation, functional status, laboratory findings, and medications. The study data were collected on admission and throughout the hospital course by the expert registry team.

Definitions of the all variables registered from the patients, outcome parameters as well as the diagnosis of disease entity like cardiogenic shock, ACS infective endocarditis, and cardiomyopathies, were carried out following the American College of Cardiology (ACC) clinical data standards [7]. Valvular heart disease was defined as moderate to severe aortic and/or mitral valve disease with or without a previous history of valvular surgery, while hypertensive heart disease was defined as the presence of concentric left ventricular hypertrophy (mean thickness of the ventricular septum and LV posterior wall ≥ 12 mm) in patients with a history of or receive treatment for hypertension.

Clinical characteristics, risk factors, and previous history were determined. All included patients are symptomatic functional class (NYHA) II–IV. In the emergency department, the handling physician diagnosed HF within 30 min of admission (depending on the described criteria) by filling out a patient standard report form. HF was defined as new-onset HF or acute decompensation of chronic HF with symptoms that were sufficient to warrant hospitalization [7].

The Charlson comorbidity index (CCI) [7] is a method that predicts mortality by classifying or weighting comorbidities like stroke, renal disease, liver disease, and cancer. It is an intensive care index utilized by health researchers to assess disease burden and considered to be an applicable prognostic indicator for mortality.

### Outcomes

The status of registered patients was surveyed during hospitalization; the following information was obtained: duration of hospital stay, death, and patient destination after discharge (home or ward).

#### Statistical analysis

All demographic, clinical characteristics, medications, and intervention were compared using *χ*^2^ test for categorical variables and unpaired *t* test for continuous variables. The relationship between gender and hospital outcomes was assessed using logistic regression analysis. Cox proportional hazard modeling was employed to all-cause mortality during hospital course. Recorded data were analyzed using the Statistical Package for Social Sciences, version 20.0 (SPSS Inc., Chicago, Illinois, USA). Quantitative data were expressed as mean ± SD. Qualitative data were expressed as frequency and percentage, and *P* value less than 0.05 was considered significant. Adjusted outcomes were presented as hazard ratio (HR) with 95% confidence interval (CI).

## Results

Baseline characteristics of women and men with HF, women with and without HF, and comparison of women with HFrEF and HFpEF are listed in Tables 1, 2, and 3. Among the 1006 patients admitted to CCU in 1 year, 345(34.2%) patients were females and 118 (34.2%) had evidence of HF, whereas 661 (65.7%) were males; 178 (26.9%) of them had HF. Regarding the HF type, in women, 73 (61.9%) had HFrEF versus 113 (63.5%) in men, *P* = 0.345, while 45 (38.1%) had HFpEF versus 65 (36.5%) in men, P = 0.378.

**Table 1:** Characteristics of HF according to gender

	Male, *n* = 178	Female, *n* = 118	*P* value
HFrEF	113 (63.5%)	73 (61.9%)	0.579
Age	59.9 ± 9	65.3 ± 11	0.0001
BMI	29.84 ± 3.2	31.63 ± 4	0.01
Obesity	81 (54.5%)	80 (67.8%)	0.000
DM	74 (41.6%)	45(38.1%)	0.628
HTN	109 (61.2%)	67(56.8%)	0.470
Dyslipidemia	59 (33.1%)	47(39.8%)	0.266
Smoking	120 (67%)	2 (1.7%)	0.000
Previous MI	52 (29.6%)	19 (16.1%)	0.019
Previous PCI	34 (19.1%)	12 (10.2%)	0.043
Prior CABG	18 (10.1%)	8 (6.78%)	0.226
Valve surgery	8 (4.5%)	6 (5.1%)	0.339
Addiction	13 (7.3%)	0 (0%)	0.001
STEMI	22 (12.4%)	16 (14%)	0.329
UA/NSTEMI	16 (9%)	21 (8.5%)	0.878
Cardiogenic shock	39 (22%)	25 (21.2%)	0.351
CHB	3 (1.7%)	1 (0.8%)	0.296
AF/Flutter	16 (8.9%)	25 (21.2%)	0.041
IE	1 (0.6%)	1 (0.8%)	0.916
PE	1 (0.6%)	3 (2.5%)	0.148
Aortic dissection	1 (0.6%)	3 (2.5%)	0.148
Cardiomyopathies	18 (10.1%)	23 (19.5%)	0.011
Chest pain (CP)	35 (19.7%)	25 (21.2%)	0.199
Orthopnea	118 (66%)	80 (67.8%)	0.195
PND	40 (22.5%)	16 (13.6%)	0.040
Palpitations	18 (10.1%)	14 (11.9%)	0.191
Syncope	10 (5.62%)	1 (0.85%)	0.024
Cough	55 (30.9%)	38 (32.2%)	0.704
Edema	63 (35.4%)	38 (32.2%)	0.847
Pacemaker	5 (2.8%)	2 (1.7%)	0.320
ICD	0 (0%)	1 (0.85%)	0.163
Killip class			
I	33 (18.5%)	11 (5.9%)	0.023
II	8 (4.5%)	6 (5.1%)	0.211
III	32 (18%)	27(22.8%)	0.119
IV	102 (57%)	71 (60.2%)	0.176
Pricardiocentesis	3 (1.7%)	1 (0.85%)	0.541
Thazides	22 (12.4%)	8 (6.8%)	0.119
Loop diuretics	109 (61%)	68(57.6%)	0.535
Nitrates	103 (58%)	67 (56.8%)	0.853
Warfarin	63 (35.4%)	31 (26.3%)	0.099
Clopedogril	75 (42.1%)	58 (49.2%)	0.235
Aldesterone antagonist	88 (49.4%)	50 (42.4%)	0.233
Digixon	67 (37.6%)	32 (27.1%)	0.060
Duration of stay	8.71 ± 7	7.06 ± 5	0.020
Mortality	19 (10.7%)	13(11.1%)	0.740
HFpEF	65 (36.5%)	45(38.1%)	0.524
Sepsis	1 (0.6%)	3(2.5%)	0.254
Pneumonia	5 (2.81%)	6 (5.1%)	0.204
AKI	23 (13%)	12 (10.2%)	0.336
CKD/ESRD	7 (4%)	3 (2.5%)	0.291
Resp. failure	0(0%)	4(3.4%)	0.016
liver failure	1(0.6%)	9(7.6%)	0.000
Tamponade	1 (0.6%)	3 (2.5%)	0.254
GIT bleeding	1 (0.6%)	3 (2.5%)	0.148
CCI	167 (93%)	112 (94%)	0.692
Previous MI	54 (30.3%)	17 (14.4%)	0.003
Previous CHF	85 (47.8%)	42 (35.6%)	0.054
PVD	5 (2.8%)	2 (1.7%)	0.296
CVA/TIA	9 (5.1%)	10 (8.5%)	0.167
Hemiplegia	1 (0.6%)	2 (1.7%)	0.220
COPD	15 (8.4%)	10 (8.5%)	0.351
VHD	14 (7.8%)	18(15.2%)	0.020
Peptic ulcer	0 (0%)	1(0.85%)	0.163
Cancer	0 (0%)	1 (0.85%)	0.163
Depression	1 (0.6%)	2 (1.7%)	0.220
Dementia	6 (3.4%)	3 (2.5%)	0.328
Metastasis	1 (0.6%)	3 (2.5%)	0.148
Endotracheal intubation	27 (15.2%)	15 (13%)	0.553
HIV	1 (0.6%)	1 (0.85%)	0.336
Cellulitis	1 (0.6%)	9 (7.6%)	0.000
PCI	20 (11.2%)	7 (6%)	0.021
CRT	3 (1.68%)	2 (1.69%)	0.329
Mechanical ventilation	28 (15.7%)	15 (22%)	0.471
CVP	121 (68%)	71 (60%)	0.168
CA	4 (2.25%)	5 (4.24%)	0.329
DC shock	8 (4.5%)	1 (0.85%)	0.044
Coma	1 (0.6%)	0 (0%)	0.437
Fever	1 (0.6%)	1 (0.85%)	
Hemoptysis	0 (0.6%)	1 (0.85%)	
Lanoxin toxicisty	0 (0%)	1 (0.85%)	
Mechanical ventilation	28 (15.7%)	15(21.7%)	0.471
Amiodarone	17 (9.6%)	7 (14.4%)	0.200
UFH	62(34.8%)	34(28.8%)	0.279
LMWH	100(56.2%)	79 (67%)	0.064
Lytic therapy	45 (25.3%)	32(27.1%)	0.724
ACEI/ARBS	112(62.9%)	78(66.1%)	0.741
Warfarin	22 (12.4%)	22(18.6%)	0.137
Beta-blockers	61 (34.3%)	45 (38%)	0.794
Survivors	159 (89%)	105 (88%)	0.750

*BMI* body mass index, *DM* diabetes mellitus, *HTN* hypertension, *STEMI* ST segment elevation myocardial infarction, *UA* unstable angina, *IE* infective endocarditis, *PE* pulmonary embolism, *PND* paroxysmal nocturnal dyspnea, *CHB* complete heart block, *Af* atrial flutter, *AF* atrial fibrillation, *CCI* Charlson comorbidity index, *PCI* percutaneous coronary intervention, *CA* coronary angiography, *CVA* cerebrovascular accident

**Table 2:** Clinical characteristics and co morbidities of females with and without HF

	No HF, *N* = 277	HF, *N* = 118	*P* value		No HF, *N* = 277	HF, *N* = 118	*P* value
Clinical data and etiology				Associated comorbidities			
Age	49.8 ± 14.5	60.3 ± 10.5	0.000	Sepsis/shock	5 (1.8%)	3 (2.5%)	0.049
BMI	30.35 ± 3.1	31.6 ± 4	0.000	Pneumonia	5 (1.8%)	6 (5.1%)	0.025
Obesity	150 (54.5%)	80 (67.8%)	0.012	AKI	20 (7.22%)	12 (10.17%)	0.114
DM	125 (41.6%)	45 (38.1%)	0.199	CKD/ESRD	6 (2.2%)	3 (2.5%)	0.136
HTN	121 (61.2%)	67 (56.8%)	0.017	Respir failure	2 (0.72%)	4 (3.4%)	0.019
Dyslipidemia	86 (33.1%)	47 (39.8%)	0.091	Liver failure	1 (0.36%)	9 (7.6%)	0.000
Smoking	153 (67.4%)	2 (1.7%)	0.619	Tamponade	9 (3.25%)	3 (2.5%)	0.021
Previous MI	10 (3.6%)	19 (16.1%)	0.000	GIT bleeding	1 (0.36%)	3 (2.5%)	0.047
Previous PCI	10 (3.6%)	12 (10.2%)	0.004	Previous MI	6 (2.2%)	17 (14.4%)	0.000
Previous CABG	3 (1.1%)	8 (6.78%)	0.001	Prior CHF	3 (1.1%)	42 (35.6%)	0.000
Valve surgery	0 (0%)	6 (5.1%)	0.000	PVD	3 (1.1%)	2 (1.7%)	0.123
Addiction	3 (7.3%)	0 (0%)	0.256	CVA/TIA	5 (1.8%)	10 (8.5%)	0.001
STEMI	55 (20%)	16 (14%)	0.053	Hemiplegia	1 (0.36%)	2 (1.7%)	0.052
UA/NSTEMI	74 (26.7%)	21 (8.5%)	0.000	COPD	14 (5.1%)	10 (8.5%)	0.056
Cardiogenic shock	0 (0%)	25 (21.2%)	0.000	DM/end organ damage	7 (2.5%)	8 (6.8%)	0.017
CHB	32 (11.6%)	1 (0.8%)	0.000	Mild liver dis	5 (1.8%)	1 (0.85%)	0.111
AF/Flutter	25 (9%)	22 (18.6%)	0.007	Severe liver dis	2 (0.72%)	1 (0.85%)	0.139
IE	8 (3%)	1 (0.8%)	0.000	peptic ulcer	0 (0%)	1 (0.85%)	0.043
PE	79 (28.5%)	3 (2.5%)	0.000	Cancer	12 (4.33%)	1 (0.85%)	0.031
Aortic dissection	1 (0.4%)	3 (2.5%)	0.047	Metastasis	8 (2.88%)	3 (2.5%)	0.26
HR (b/min)	108.9 ± 38	110 ± 37	0.758	Dementia	0 (0%)	3 (2.5%)	0.004
SBP (mmHg)	128.7 ± 29	121.54 ± 37	0.03	Autoimmu. D	13 (4.7%)	3 (2.5%)	0.009
DBP (mmHg)	80.2 ± 18	75.3 ± 20.5	0.01	CCI	277 (100%)	112 (94.9%)	0.000
Hospital stay (days)	7.14 ± 6	7.06 ± 5	0.892	Mortality (%)	14 (5.1%)	13 (11.1%)	0.041

*BMI* body mass index, *DM* diabetes mellitus, *HTN* hypertension, *STEMI* ST segment elevation myocardial infarction, *UA* unstable angina, *IE* infective endocarditis, *PE* pulmonary embolism, *CHB* complete heart block, *Af* atrial flutter, *AF* atrial fibrillation, *PCI* percutaneous coronary intervention, *CA* coronary angiography, *CVA* cerebrovascular accident, *HR* heart rate, *SBP* systolic blood pressure, *DBP* diastolic blood pressure, *CCI* Charlson comorbidity index

**Table 3:** Clinical characterestics, co-morbidities and medications in HFrEF and HFpEF subgroups

	HFrEF, *n* = 73	HFpEF, *n* = 45	*P* value
Age (years)	66.9 ± 9	52.3 ± 11	0.0001
Chest pain (CP)	22 (30%)	1 (2.22%)	0.001
Killip class			
I	7 (9.6%)	4 (9%)	0.935
II	4 (5.5%)	2 (4.44%)	0.915
III	16 (22%)	11 (24.44%)	0.887
IV	44 (60.3%)	27 (60%)	0.941
Orthopnea	50 (68.5%)	30 (66.67%)	0.932
PND	12 (16.4%)	4 (9%)	0.488
Palpitations	3 (4.12%)	11 (24.44%)	0.004
Syncope	1 (1.4%)	0 (0%)	0.692
Cough dry	3 (4.12%)	5 (11.11%)	0.142
Productive cough	19 (26%)	11 (24.44%)	0.339
Edema	26 (35.6%)	12 (26.67%)	0.256
Previous MI	13 (17.8%)	6 (13.33%)	0.500
Previous PCI	9 (12.3%)	3(6.67%)	0.380
Previous CABG	7 (9.6%)	1(2.22%)	0.190
Valve surgery	1 (1.4%)	5(11.11%)	0.035
STEMI	16 (21.9%)	0 (0%)	0.002
STEMI	16 (21.9%)	0 (0%)	0.002
UA/NSTEMI	8 (11%)	13(28.9%)	0.000
CHB	1 (1.34%)	0 (0%)	0.436
AF/flutter	10 (13.7%)	12 (26.6%)	0.039
IE	0 (0%)	1 (2.2%)	0.077
PE	1 (1.34%)	2 (4.44%)	0.303
Aortic dissection	1 (1.34%)	2 (4.44%)	0.303
Hypertension	4(5.5%)	11(24.44%)	0.000
Prior HF	20 ( 27%)	8 (17.78%)	0.000
RHD	0 (0%)	4 (8.89%)	0.000
Pacemaker	1 (1.4%)	2 (4.44%)	0.108
ICD	1 (1.4%)	0 (0%)	0.436
CVP	52 (71.2%)	19 (42.2%)	0.002
Endotracheal intubation	15 (20.5%)	0 (0%)	0.001
Thazides	22 (12.4%)	8 (6.8%)	0.119
Loop diuretics	109 (61%)	68 (57.6%)	0.535
Nitrates	103 (58%)	67 (56.78%)	0.853
Warfarin	63 (35.4%)	31 (26.3%)	0.099
Clopedogril	75 (42.1%)	58 (49.2%)	0.235
Hospital stay	7.88 ± 5.7	5.73 ± 3.4	0.02
Sepsis	1 (1.4%)	2 (4.4%)	0.303
Pneumonia	4 (5.5%)	2 (4.44%)	0.575
Fever	0 (0%)	1 (2.22%)	0.172
Hemoptesis	1 (1.4%)	0 (0.85%)	
Lanoxin toxicisty	0 (0%) 0 (0%)	1 (2.22%) 1 (2.22%)	
CKD/ESRD	3 (4.12%)	0 (0%)	0.236
Respiratory failure	2 (2.7%)	2 (4.44%)	0.511
liver failure	3 (4.12%)	6 (13.3%)	0.067
Tamponade	1 (1.4%)	2 (4.44%)	0.303
GIT bleeding	1 (1.4%)	2 (4.44%)	0.303
CCI	71 (97.3%)	41 (91%)	0.140
Previous MI	13 (17.8%)	4 (4.44%)	0.261
AKI	12 (16.4%)	0 (0%)	0.016
PVD	1 (1.4%)	1 (2.22%)	0.549
CVA/TIA	7 (9.6%)	3 (6.7%)	0.518
Hemiplegia	2 (2.7%)	0 (0%)	0.322
COPD	7 (9.6%)	3 (6.67%)	0.056
Autoimmune	8 (11%)	0 (0%)	0.046
Mild liver diseases	1 (1.4%)	0 (0%)	0.436
peptic ulcer	0 (0%)	1 (2.22%)	0.254
Cancer	1 (1.4%)	0 (0%)	0.436
Metasis	1 (1.4%)	2 (4.4%)	0.303
Dementia	1 (1.4%)	2 (4.44%)	0.336
Rheumatic D	1 (1.4%)	2 (4.4%)	0.303
HIV	0 (0%)	1 (2.22%)	0.254
Cellulitis	3 (4.12%)	6 (13.%)	0.067
Depression	0 (0%)	2 (4.4%)	0.108
Mechanical ventilation	14 (19.2%)	1 (2.22%)	0.007
Pricardiocentesis	0 (0%)	1 (2.22%)	0.201
PCI	7 (9.6%)	0 (0%)	0.032
Coronary angiography	5 (6.8%)	0 (0%)	0.073
Amiodarone	17 (9.6%)	7 (14.4%)	0.200
Unfractunated heparin	62 (34.8%)	34(28.8%)	0.279
LMWH	100 (56.2%)	79 (67%)	0.064
Lytic therapy	45 (25.3%)	32 (27.1%)	0.724
Mortality	12 (16.4%)	1 (2.22%)	0.01

*BMI* body mass index, *DM* diabetes mellitus, *HTN* hypertension, *STEMI* ST segment elevation myocardial infarction, *UA* unstable angina, *IE* infective endocarditis, *PE* pulmonary embolism, *CHB* complete heart block, *Af* atrial flutter, *AF* atrial fibrillation, *PCI* percutaneous coronary intervention, *CA* coronary angiography, *CVA* cerebrovascular accident, *HR* heart rate, *SBP* systolic blood pressure, *DBP* diastolic blood pressure

### Comparison between women and men with HF

**Fig. 2:**
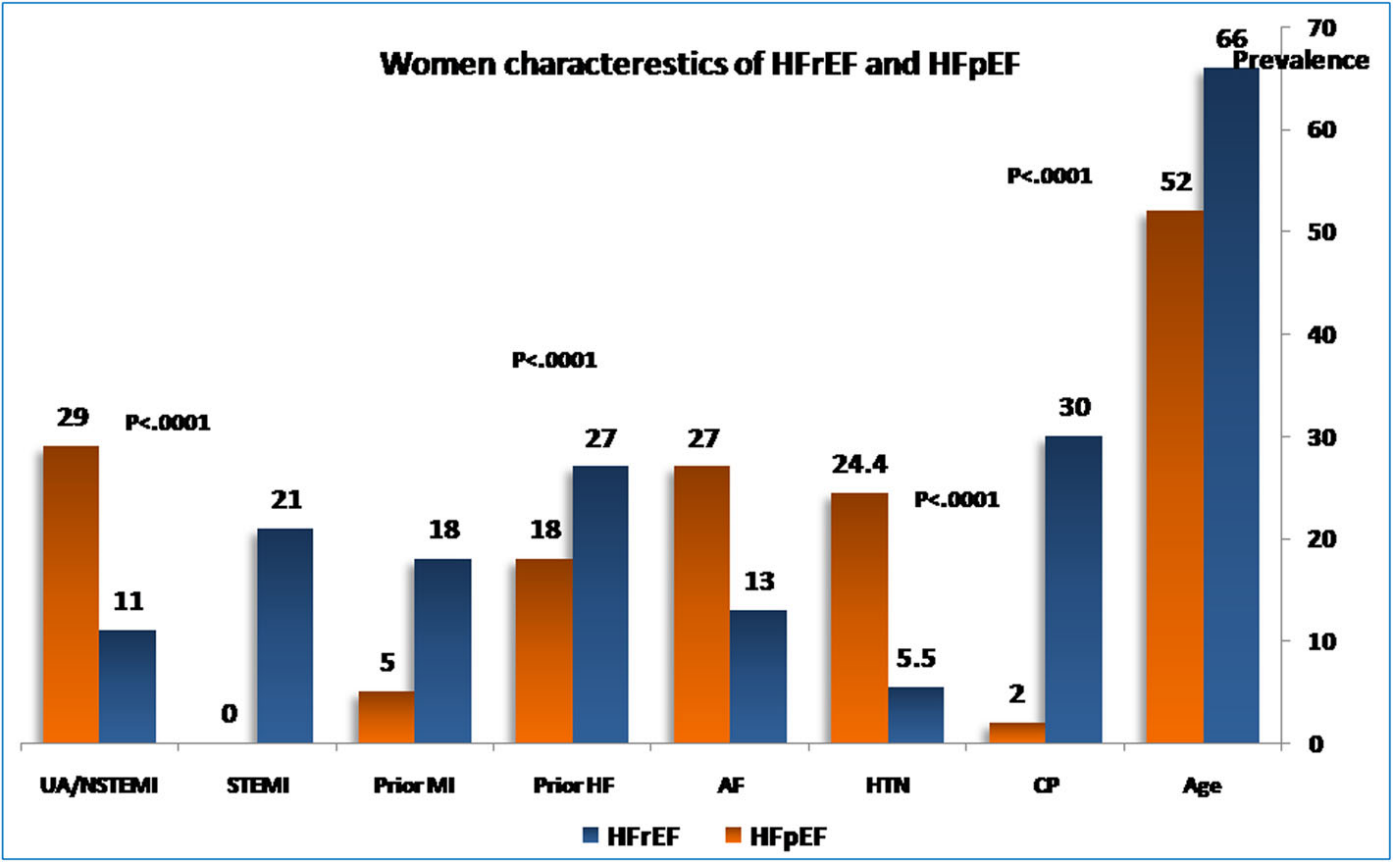
Women characteristics of HFrEF and HFpEF

Women with HF were older in age, more obese, and less symptomatic than men. Women had higher incidence of associated comorbidities like liver failure, respiratory failure, and cellulitis. On the contrary, the prevalence of smoking, addiction, and previous MI and PCI were lower in women than in men. Women are less liable to be repeatedly admitted to the hospital for HF and less likely to have ischemic heart disease as underling etiology of HF. However, valvular heart diseases (VHD), atrial fibrillation (AF), and cardiomyopathies were more likely to be the etiologies of their HF (Fig. 2).

Accordingly, with the lower prevalence of coronary heart disease, women were less likely to undergo percutaneous coronary intervention (PCI) or coronary artery bypass grafting (CABG). Meanwhile, women treated with implantable cardioverter defibrillator (ICD) and/or cardiac resynchronization therapy (CRT) and cardiac pacemakers at similar frequencies as men with HF.

Compared to men, women with HF had more normal ECG: 36 (30.5%) versus 36 (20.2%) *P* < 0.01, more prevalence of left anterior hemiblock (LAH) 5 (4.2%) versus 2 (1.1%) *P* < 0.02, AF 30 (25.4%) versus 30 (16%) *P* < 0.04, and less likely to have LBBB 15 (12.7%) versus 60 (33.7%), *P* < 0.00001. Regarding echocardiographic data, women had higher EF% 47 ± 13 versus 40 ± 13, *P* < 0.05 and smaller LA size 18.18 ± 18 versus 22.69 ± 19, *P* < 0.04; nevertheless, there was no considerable difference between women and men in grades of diastolic dysfunction, severity of mitral regurg, RWMA or E/e' (*P* = NS), or routine laboratory workup.

There was no significant difference in medications of invasive procedures like central venous pressure (CVP), endotracheal intubation, pacemakers, or ventilation prescribed during CCU admission between women and men.

**Fig. 3:**
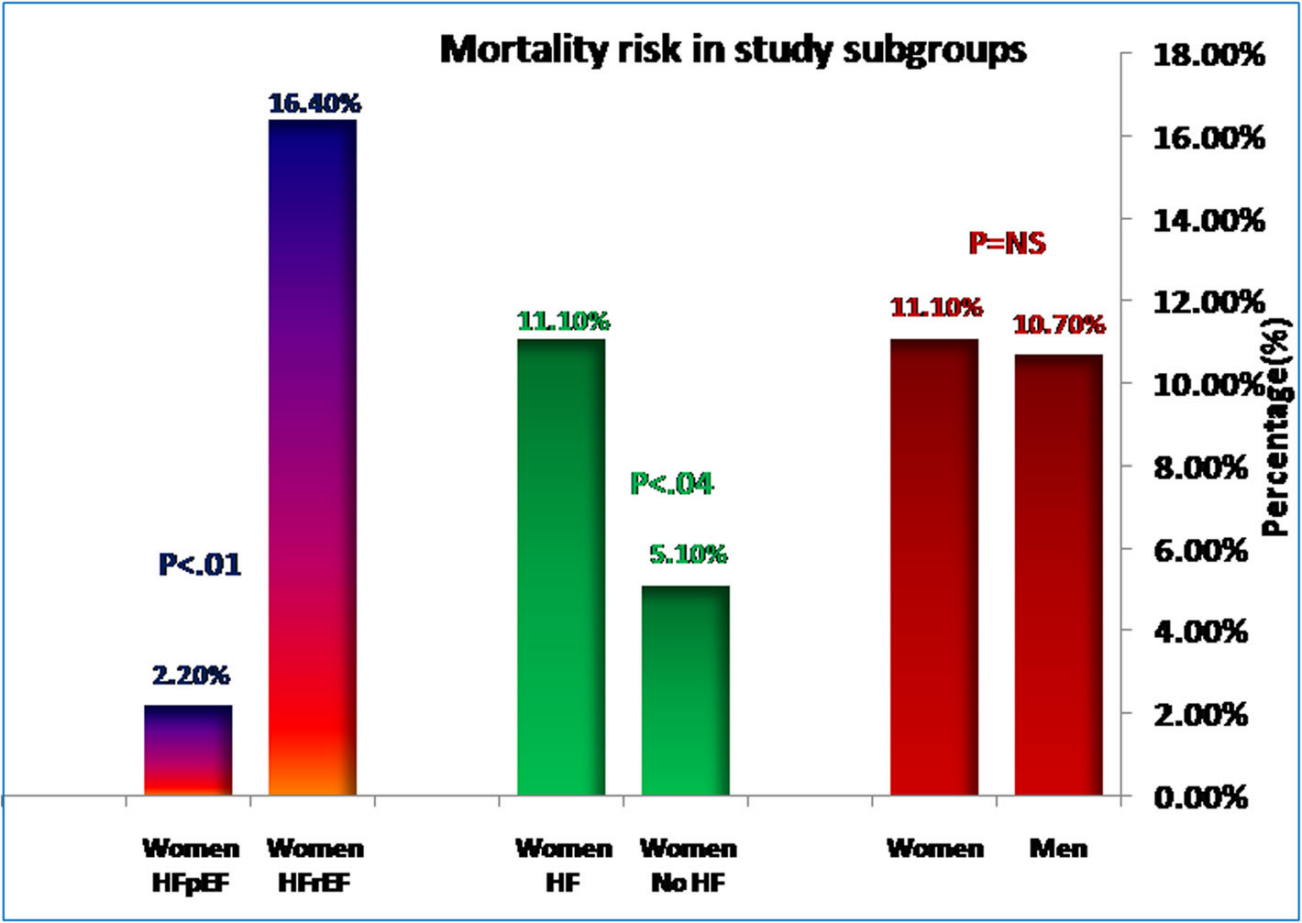
Mortality risk in study subgroups

Women with HF showed shorter stay in CCU compared to men. The mortality risk during hospitalization did not differ by gender (Fig. 3).

### Comparison between women with and without HF

Comparing the 118 females with HF to 277 patients without HF (Table 2), HF females were older, more obese with higher BMI, had prevalent prior MI, with more PCI, CABG, and valve surgery. Females with HF had a higher prevalence of STEMI, NSTEMI/UA, pulmonary embolism (PE), infective endocarditis (IE), and aortic dissection and higher incidence of significant arrhythmias like AF and CHB. More hemodynamic compromise is recorded in HF female’s subgroup including higher heart rate and more hypotension. Additionally, women with HF had more frequent associated comorbidities, hepatic diseases, GIT bleeding, CVD, dementia, respiratory failure, peptic ulcer, and pneumonia. However, non-HF women had higher prevalence of cancer and autoimmune diseases.

On ECG, women with HF had higher prevalence of voltage criteria 14 (11.9%) versus 1 (0.44%), *P* < 0.0001, AF [30(25%) versus 11(4%)] *P* < 0.0001 pathologic Q wave 36 (30.5%) versus 62 (22.4%) *P* < 0.0001 compared to non-HF subgroup.

Regarding laboratory workup, women with HF had higher LDL level (154.15 ± 38 versus 140.88 ± 33 mg/dl, *P* < 0.01), FBS (209.66 ± 145 versus 149.76 ± 108 mg/dl, *P* < 0.001) higher A1c level 9.03 ± 2 versus 7.83 ± 3, *P* < 0.001, higher creatinine level (2.24 ± 3.2 versus 1.41 ± 1 mg/dl), ALT (65.7 ± 68 versus 42.41 ± 34 u, P < 0.0001) and higher INR ratio (1.54 ± 1 versus 1.21 ± 1, mg/dl, *P* < 0.001), lower hemoglobin (10.96 ± 3 versus12.00 ± 1, gm/dl *P* < 0.0001) and albumin (3.86 ± 1 versus 4.03 ± 1 mg/dl, *P* < 0.001.

The higher risk profile of women with HF is associated with increased mortality risk despite similar duration of hospital stay.

### HFrEF and HFpEF in women

Unexpectedly, HFrEF was the commonest type of HF 73 (61.9%) versus HFpEF 45 (38.1%) in females (*P* < 0.001); the averaged value of EF was 33.88% in patients with reduced EF, while it was within the normal range for patients with preserved EF (61.4%) (Table 4). Comparing patients with reduced EF, to patients with preserved EF, they were significantly younger, had prevalent hypertension, more UA/NSTEMI, less STEMI and CABG, and less valve surgery. The causes of HF were ACS in a larger percentage of patients with reduced EF, where hypertensive heart disease and valvular HD were more common in those with preserved EF. Patients with reduced EF were also more likely to have frequent admission to hospital with CHF and more comorbidities like acute kidney injury (AKI) and COPD, while no difference in medications prescribed by CCU physicians between the two types. In contrast, mechanical ventilation, pacemakers, and CVP were higher in HFrEF. Clopidogrel, proton pump inhibitors (PPIH) and aspirin (ASA) were more commonly prescribed to HFrEF while calcium channel blockers (CCB) were more frequently prescribed to HFpEF (Table 5).

**Table 4:** ECHO findings in female HF types

		Mean	*P* value
LVEDd (mm)	HF-REF	39.07 ± 25	0.004
	HF-PEF	17.53 ± 22	
LVESd (mm)	HFrEF	23.84 ± 21	0.025
	HFpEF	15.05 ± 20	
IVS (mm)	HFrEF	5.42 ± 5	0.021
	HFpEF	3.40 ± 4	
PWT (mm)	HFrEF	5.36 ± 5	0.018
	HFpEF	3.30 ± 4	
EF%	HFrEF	33.88 ± 12	0.001
	HFpEF	61.96 ± 12	
LA (mm)	HFrEF	21.50 ± 19	0.012
	HFpEF	12.87 ± 16	
AO (mm)	HFrEF	28.26 ± 16.5	0.015
	HFpEF	10.76 ± 15	
TAPSE (mm)	HFrEF	6.84 ± 5.1	0.235
	HFpEF	8.65 ± 4	
EPASP (mm)	HFrEF	42.26 ± 13	0.503
	HFpEF	40.12 ± 20	
E/A wave	HFrEF	1.47 ± 1	0.409
	HFpEF	1.67 ± 1	
E/e' wave	HFrEF	18.31 ± 9.5	0.023
	HFpEF	23.62 ± 12	
RWMA	HFrEF	62 (85%)	0.002
	HFpEF	27 (60%)	

*HFrEF* heart failure with reduced ejection fraction, *HFpEF* heart failure with preserved ejection fraction, *LVEDd* left ventricular end diastolic diameter, *LVESd* left ventricular end systolic diameter, *IVS* interventricular thickness, *PWT* posterior wall thickness, *EF%* ejection fraction, *LA* left atrium, *Ao* aortic root diameter, *TAPSE* tricuspid annular plane systolic excursion, *EPASP* estimated pulmonary artery systolic pressure, *E?A* mitral flow early diastolic velocity, *A* mitral inflow atrial diastolic velocity, *E/e* mitral flow early diastolic velocityto early diastolic mitral annular velocity, *RWMA* regional wall motion abnormalities

**Table 5:** Medications in women with HFrEF and HFpEF subgroups

	HFrEF, *n* = 73	HFpEF, *n* = 45	*P* value
Thazides	5 (6.85%)	3 (6.7%)	0.969
Loop diuretics	40 (54.8%)	28 (62.22%)	0.428
Nitrates	39 (53.4%)	28 (62.22%)	0.349
Warfarin	17 (23.3%)	14 (31.11%)	0.348
Clopidogril	41 (56.2%)	17 (37.8%)	0.052
CCB	2 (2.74%)	6 (13.33%)	0.026
Amiodarone	10 (13.7%)	7 (15.6%)	0.780
Unfractunated heparin	19 (26.02%)	15 (33.33%)	0.395
LMWH	52 (71.23%)	27 (60%)	0.208
Lytic therapy	24 (32.88%)	8 (17.8%)	0.073
Aldosterone antagonist	32 (43.8%)	18 (40%)	0.682
Digixon	19 (26.03%)	13 (28.9%)	0.734
PPIH blockers	55 (75.34%)	42 (93.3%)	0.013
Warfarin A	10 (13.7%)	12 (26.7%)	0.079
ASA	60 (82.2%)	28 (62.22%)	0.016
Beta-blockers	27 (37%)	18 (40%)	0.743
ACEI	38 (52.1%)	22 (49%)	0.738
ARBs	8 (11%)	10 (22.22%)	0.098
Statin	58 (79.5%)	32 (71.1%)	0.301

*CCB* calcium channel blockers, *LMWH* low molecular weight heparin, *ASA* acetyl salsylic acid, *ACEI* angiotensin converting enzyme inhibitor, *ARBS* angiotensin receptor blockers

Regarding ECG changes, women with HFpEF had higher prevalence of voltage criteria, 13 (28.9%) versus 1 (0.4%) in HFrEF, *P* < .00001, but lower frequency of pathologic Q and ischemic changes, 14 (31%) versus 32 (43.8%) in HFrEF, *P* < 0.0001.

Patients with HFpEF illustrated shorter duration of hospital stay compared with those with HFrEF. However, HFrEF showed higher risk of mortality compared to HFpEF. Mortality was significantly higher in HFrEF 12% versus 1% in HF with HFpEF.

## Discussion

In the current study, the main findings are that substantial gender differences exist among Egyptian HF patients; women with HF are older, more obese, less smoker, and have more comorbidities, and HFrEF is the commonest type. Valvular heart diseases and cardiomyopathies are commonest etiology of HF. Female HF patients have similar survival during hospital course compared with men with HF.

### Gender difference in clinical characteristics in CCU HF patients

The present study demonstrated gender differences in patients’ clinical characteristics, comorbidities, and short-term outcome who were admitted to CCU. Female patients were characterized by older age, higher LVEF, lower prevalence of ACS, larger incidence of valvular heart disease, and cardiomyopathies in our study which is consistent with previous reports [8, 9].

The clinical manifestations of HF appeared to be less severe in women compared with men, and women had lower NYHA functional class but similar laboratory workup despite the higher and preserved LVEF%. Treatment according to latest guidelines, however, was equally or even similar to that given to women compared with men. This is in contrast to previous reports from Japanese registry [7].

### Women in CCU HF patients

While previous studies of HFpEF reported that the female sex is dominant in patients with HFpEF [10], in the current study, the proportion of female to male in HFpEF was almost the same. The proportions of the females were 42% in the Japanese Diastolic Heart Failure Study (JDHF) [11] and 45% in the Japanese Cardiac Registry of Heart Failure in Cardiology (JCARE-CARD) [12], both studies enrolled Japanese patients. However, in the current registry, the proportion of HFpEF in female HF patients was similar to men: 73 (61.9%) had HFrEF versus 113 (63.5%) in men, *P* = 0.345 while 45(38.1%) had HFpEF in women versus 65 (36.5%) in men, *P* = 0.378.

The clinical characteristics of the study population were almost comparable to those of the ADHERE and OPTIMIZE-HF [6, 7]. HF patients with reduced EF were older, more obese, and more likely to have ACS. They were less likely to have a hypertension or valvular HD.

Higher prevalence of HFrEF in female patients most likely reflects the impact of age on cardiac structure and the high prevalence of coronary artery disease in this group of HF patients. HFpEF female patients had higher prevalence of hypertension and atrial fibrillation, which may possibly be a consequence as well as a causative factor for clinical presentation of such type of HF.

Additionally, respiratory failure, hepatic failure, cellulites, anemia, and hypo-albuminemia are the common comorbidities compared to men. Anemia is a strong predictor of mortality and morbidity in HF patients [5, 9], also appeared to be highly prevalent in our study, might be explained by renal dysfunction, older age, and more obesity that seems to explain the large discrepancy.

Basic HF therapy in the form of diuretics, renin-angiotensin system (RAS) inhibition, ACE-inhibitors, or angiotensin receptor blockers in 66% and aldosterone antagonist in 42% were similarly utilized in both women and men. These percentages are noticeably higher compared to Asian AHF registry [10] where ACEI/ARBS used in only 37% and spironolactone in 34%. Actually, the worse renal function in Asian registry (creatinine clearance 69 ml/min in this young population) could be an explanation. In accordance to our registry, the lower use of beta-blockers in 34% and 24% in Asian registry is considered and might reflect the concern of worsening HF in patients with already advanced syndrome. Digoxin and nitrates are easily available drugs traditionally but employed modestly in women with HF, it may reflect slower adoption of contemporary HF management and/or lower cost of these drugs [13].

### Gender difference in short-term prognosis

One of the main findings of the present study is the similar mortality rate during CCU admission in both gender despite the difference of HF etiology, the clinical presentation, patients risk profile, and comorbidities. Our findings are confirmed by the Sakata et al. [8] in their report from the CHART-2 study, and they examined the gender difference in long-term outcome in 4736 consecutive CHF patients and found that the incidence of mortality and other events in women and men with stage C/D HF experienced 52.4 and 47.3 deaths per 1000 person-years (*P* = 0.225) and 58.3 and 51.3 cases of HF requiring admission per 1000 person-years (*P* = 0.189), respectively. They concluded that there were no gender differences in all-cause death and HF requiring admission, although the incidences of both events are much higher than those of AMI or stroke [12]

## Limitations

The present study had following limitations. First, this was a single-center study involving a relatively small number of HF patients that included both gender and both types of HF (HFrEF and HFpEF) patients. Second, our registry population was limited to patients who were admitted to the CCU; HF patients who were admitted to general wards were excluded from this study. Third, this study was designed for short term outcome and had a relatively no follow-up period like those in previous reports.

## Conclusion

In conclusion, although female patients had different clinical characteristics and underlying etiologies of HF which is varied from male gender, their short-term outcome and hospital mortality are similar. HF with reduced ejection fraction was present in a considerable proportion of hospitalized female patients admitted to CCU in unselected critically ill stage and associated with higher mortality risk compared to HFpEF. Given the higher risk of adverse clinical events and the lack of a satisfactory proof to guide the treatment, clinical trials are critically required to identify the effective preventive strategies for women with HF.

## Data Availability

The dataset supporting the results and conclusions of this article will be available from the corresponding author on request.

## Abbreviations

CCU: Cardiac care unit
HF: Heart failure
HFpEF: Heart failure with preserved ejection fraction
HFrEF: Heart failure with reduced ejection fraction
ACS: Acute coronary syndrome
